# Serve Size and Estimated Energy and Protein Contents of Meals Prepared by ‘Meals on Wheels’ South Australia Inc.: Findings from a Meal Audit Study

**DOI:** 10.3390/foods7020026

**Published:** 2018-02-20

**Authors:** Tony Arjuna, Michelle Miller, Stijn Soenen, Ian Chapman, Renuka Visvanathan, Natalie D Luscombe-Marsh

**Affiliations:** 1 National Health and Medical Research Council (NHMRC) Centre of Research Excellence in Translating Nutritional Science to Good Health, Discipline of Medicine, The University of Adelaide, Adelaide 5005 Australia; tony.arjuna@ugm.ac.id (T.A.); stijn.soenen@adelaide.edu.au (S.S.); ian.chapman@adelaide.edu.au (I.C.); renuka.visvanathan@adelaide.edu.au (R.V.); 2 Department of Nutrition and Health, Faculty of Medicine, Universitas Gadjah Mada, Yogyakarta 55281, Indonesia; 3 Department of Nutrition and Dietetics, School of Health Sciences, Flinders University, Bedford Park 5042, Australia; michelle.miller@flinders.edu.au; 4 Adelaide Geriatrics Training and Research with Aged Care (G-TRAC) Centre, National Health and Medical Research Council Centre of Research Excellence Frailty Trans-Disciplinary Research to Achieve Healthy Ageing, Discipline of Medicine, The University of Adelaide, Adelaide 5005, Australia; 5 Aged and Extended Care Services, the Queen Elizabeth Hospital, Central Adelaide Local Health Network, Adelaide, Woodville South 5011, Australia; 6 Nutrition and Health Program, Health and Biosecurity Business Unit, Commonwealth Scientific Industrial Research Organization (CSIRO), Adelaide 5000, Australia

**Keywords:** meals on wheels, nutrition, community, meal service, ageing

## Abstract

An audit of ‘standard’ (STD) and ‘energy and protein fortified’ (HEHP) meals from Meals on Wheels (MOW) South Australia’s summer menu was conducted to evaluate the consistency, and serve size and nutrient contents, of their menu items. Twenty soups, 20 mains and 20 desserts from each of the STD and HEHP menus were prepared at the MOW South Australia’s kitchen and delivered to three ‘sham(dummy)-clients’ over a 5-week period. Each meal component was weighed in triplicate, to the nearest gram, the variation within the serve weight was calculated, and the overall energy and protein content of each meal was determined using FoodWorks (Xyris Software, Highgate Hill, Queensland, Australia). On average, the variability for soups and mains was ≤6% and for desserts was ≤10% and although the measured serve sizes of the MOW meals were consistently smaller than prescribed serve size, the differences were minor. As a percentage of recommended daily intakes (RDIs) for adults aged over 60 years, we calculated that the STD meals contained 21–39% for energy and 42–63% for protein while the HEHP meals contained 29–55% for energy and 46–69% for protein. These findings demonstrate that MOW meals currently meet the voluntary meal guidelines for energy and protein.

## 1. Introduction

Governments world-wide recognise that adequate nutrition is fundamental for the maintenance of health, independence and the quality of life of people across all life-stages. The provision of optimal nutrition for our ageing populations—especially for the 900 million who are currently aged ~60 years and older—is a key preventative health strategy for the World Health Organisation [1,2].

To assist nutritionally vulnerable adults aged ~60 years and older (and their carers) to remain living independently in the community, many countries, including the United Kingdom, Australia, the United States, Ireland and Canada, provide free or heavily government subsidised meals to their communities through programs such as ‘Meals on Wheels’ (MOW) [3,4,5]. In Australia, the MOW program operates across all states and territories representing over 600 different meal services [6]. To be eligible for funding, each meal service must abide by regulatory guidelines set by the Commonwealth Home Support Program (CHSP), which includes the Home and Community Care (HACC) program. The HACC guidelines specifically state that both home- and centre-based food services must provide a minimum two courses (namely, main course and dessert) to older adults who struggle to self-fed, prepare or shop for food, and that each meal should contain the recommended food servings providing at least one-third of recommended daily intakes (RDI) for energy, half of the RDI for protein and other vitamins and minerals, and two-thirds of the RDI for Vitamin C [7,8]. Unfortunately, however, many meal services do not know the variability within the recommended portion/serve sizes of all menu items, or the nutritional content of the meals they provide, or how well those meals comply with the government guidelines. 

The latest meal audits conducted by Australian MOW, dating back to 1986, revealed that the serve sizes of meals varied on average by 56% for main meals and 44% for vegetables [9]. Furthermore, the meals contributed, on average, to 20–30% of recommended daily energy, fat, carbohydrate, fibre, calcium, thiamine, riboflavin and niacin, requirements, and 30–40% of daily requirements for protein, iron and zinc, and 40–60% for vitamin C and retinol [9]. Meal service providers in other countries including the United States, Denmark and Ireland have also reported similar levels of variation in serve sizes and nutritional content of meals delivered [10,11,12,13]. While the development and enforcement of voluntary meal guidelines could assist meal services to deliver meals that are more consistent, both in terms of portion size and nutrient quality, and much of the variation observed during meal audits across numerous kitchens appears to be due to variation in the level of education and/or enforcement of staff to adhere strictly to recipes, and/or due to the use of non-standardised equipment, during meal preparation [6,14].

The main aim of this study was to establish the consistency, and serve size and energy and protein content, of 120 MOW menu items (60 from the standard menu and 60 from the energy and protein fortified menu) that were prepared for MOW recipients in South Australia during the summer of 2011/12. We also determined how well the meals met the HACC recommendations for older adults, especially for energy and protein. 

## 2. Materials and Methods

Sixty ‘standard’ (STD) and 60 ‘energy and protein fortified’ (HEHP) menu items (i.e., 20 soups, 20 main meals and 20 desserts) were audited over a 5-week period from late December 2011 to early February 2012 to determine the consistency of serve sizes, and the energy and protein content of the meals. The STD and HEHP meals (all 3-course hot lunchtime meals) were selected as test meals for the study too because they are the most frequently ordered meals. 

Details regarding the recommended weight of each food component within the soup, main meal and dessert menu items, as well as the overall recommended weight (i.e., serve size) of each item, are shown in Table 1. HEHP menu items were created by fortifying the STD menu items with cream, butter, semi-matured cheese and larger serve of meat. 

All menu items were cooked and packed by trained chefs who were full time employees of the MOW South Australia Kent Town kitchen facility. To ensure the MOW staff responsible for plating the meals were unaware that an audit was being conducted, all menu items were ordered in triplicate on three separate occasions by a ‘dummy client’ (i.e., Nutrition and Dietetics students from Flinders University). The usual ordering process was followed yet the meals were delivered to the homes of the ‘dummy clients’; hence, each day the students received all menu components in triplicate for the scheduled STD and HEHP three-course lunchtime meals. 

**Figure 1:**
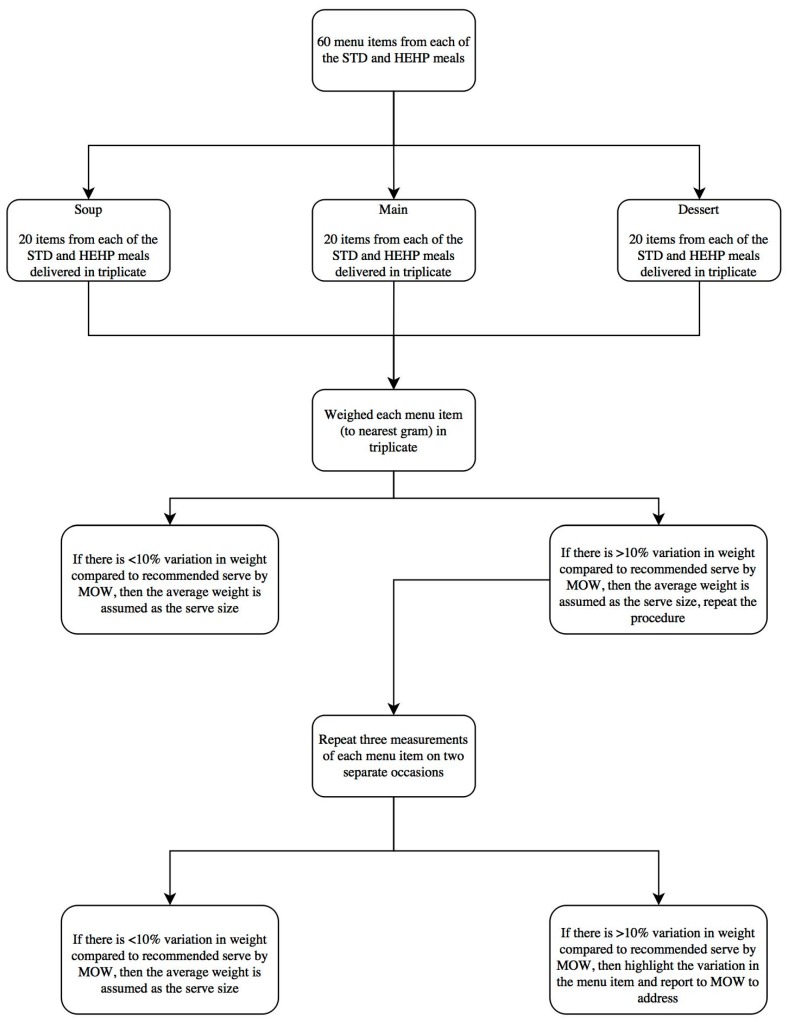
Meal audit process. STD: standard meals, HEHP: high energy and protein fortified meals; nutrient content was analyzed using FoodWorks 3.01. MOW: Meals on Wheels.

Figure 1 outlines the audit process followed in this study. After receiving three serves of the same hot lunchtime meal, the students immediately photographed and recorded the total weight of each specific menu item to the nearest gram using a set of calibrated Kenwood DS607 Digital Food Scales (Subang Jaya Selangor, Malaysia). To determine whether the recommended serve for each specific food component within each menu item was also being met, the students dissected, and separately recorded the weight of those specific food components. The nutrient content of each meal was analysed using FoodWorks 3.01 (Xyris Software, Highgate Hill, Queensland, Australia) and the Australian nutrient composition database [15] based on minimum cooke d quantities exclusive of bone, and the nutrient calculations also allowed for shrinkage on meat, chicken and fish. 

Data was analysed by calculating the mean (and standard deviation/SD) of the triplicate weights for each serve of each of the menus items. The variation in serve weight (termed coefficient of variation or, CV, expressed as a percentage) for each menu item was calculated by dividing the mean of the triplicate weights by the standard deviation, and multiplied by 100. Where there was <10% variation (which represented an acceptable level of variation), the average of the three measurements was assumed as the serve size. Where there was >10% variation, a complete re-audit on a separate occasion was conducted. The re-auditing occurred a maximum of two separate times, and if variation was still >10%, the item was highlighted for follow-up by MOW so that that the staff could implement a revised standard operating procedure to ensure uniform serves. For the purpose of this work, when re-auditing was conducted twice, then the mean of six separate measurements was used to determine the CV of the serve size for that menu item, whereas, if the re-auditing was conducted three times, then the mean of nine separate measurements was used. Finally, the difference for the actual measured, compared with prescribed, serve size (in grams) for each menu item was calculated to describe the variation between serve size of each of the menu items within the STD and HEHP meals.

## 3. Results

The measured weight of the soups in triplicate were very consistent (Table 2); however, both the prescribed and measured weights were greater than what is recommended by MOW meal policy guidelines that were presented in Table 1. The measured weights of the 20 STD and 20 HEHP soups were 198 ± 28 g (CV, 3%) and 211 ± 25 g (CV, 3%). These measured serve sizes were on average 67 g and 63 g less than the prescribed serve size of 265 for the STD and 274 for the HEHP, soups (Table 2). 

The measured weight of the main meals in triplicate was variable (Table 2). While the mean CV of triplicate menu items for the 20 STD mains was <10%, there were three STD mains that were found and reported to MOW as having variation >10%; i.e., (i) Ravioli Bolognaise Minced Meat (CV, 14% and, on average, the serve size was 40 g greater than the prescribed serve), (ii) Chicken Cacciatore (CV, 11% and, on average, the serve size was 31 g greater than the prescribed serve), and (iii) Lancashire Hot Pot (CV, 11%, and, on average, the serve size was 7 g greater than the prescribed serve). The measured weights of the 20 STD and 20 HEHP mains were 372 ± 48 g (CV, 6%) and 408 ± 36 g (CV, 6%). These measured main meal serve sizes were on average 63 g and 48 g less than the prescribed serve size of 435 g for STD and 456 g for HEHP, main meals (Table 2). 

The measured weight of dessert items in triplicate was variable (Table 2). While the mean CV of triplicate menu items for the 20 STD desserts was ≤10%, there were three STD desserts and one HEHP that were found and reported to MOW as having variation >10%; i.e., (i) STD cake and custard (CV, 14% and, on average the serve size was 32 g less than the prescribed serve), (ii) STD cake and custard (CV, 77% and, on average the serve size was 88 g less than the prescribed serve), (iii) HEHP custard crumble (CV, 14% and, on average the serve size was 57 g less than the prescribed serve), and (iv) HEHP panna cotta (CV, 16% and, on average the serve size was 33 g less than the prescribed serve). The measured weights of the 20 STD and 20 HEHP desserts were 188 ± 55 g (CV, 10%) and 245 ± 49 g (CV, 8%). These measured dessert serve sizes were on average 49 g and 12 g less than the prescribed serve sizes of 237 g for the STD and 257 g for the HEHP, desserts (Table 2). 

The average nutrient contents of the prescribed, as compared to measured STD and HEHP menu items within meals are reported in Table 3 main menu items, provided the greatest portion of energy and protein followed by the dessert and then soup menu items. For the measured compared to the prescribed STD and HEHP menu items, there was a significantly lower content of energy, protein and several micronutrients (Table 3).

Details on the estimated energy and protein requirements of a person aged 60 years and older, and the contribution of STD and HEHP MOW meals to a MOW client’s daily energy and protein intake is presented in Table 4. Based on the estimated energy and protein content determined from the measured menu items, it was calculated that the STD MOW meals would provide clients (who are aged >60 years with mean age typically being 70 years and older) with 21–39% of daily recommended energy requirements and 42–63% of their protein requirements, whereas the HEHP MOW meals would provide 29–55% of daily recommended energy requirements and 46–69% of protein requirements. The measured concentration of vitamin C was equivalent to 75% of the RDI for older Australian adults, and the concentrations of calcium, phosphorus, iron, sodium and fibre were equivalent to ≥50% of RDIs. However, the measured concentrations of potassium, magnesium, zinc and iodine only equated to ~30–40% of the RDIs for these minerals. 

## 4. Discussion

The present study presents the findings from a menu audit conducted on all ‘standard’ and ‘energy and protein fortified’ meals produced by MOW South Australia’s centralised kitchen facility. Our findings indicate that, on average, the variability for soups and mains was ≤6% and for desserts was ≤10%, and although the measured serve sizes of all menu items were consistently smaller than the MOW prescribed serve sizes, the differences were minor. Despite some inconsistency in serve sizes, the STD and HEHP meals meet the HACC and South Australia MOW Inc. guidelines for energy, protein, vitamin C, calcium, phosphorus, iron and fibre and fell ~10% short of the HACC criteria for potassium, magnesium and zinc [7,8].

The serve sizes reported here were comparable to those reported by Shovic et al. who in both 1991 and 1995 assessed meals produced by ten different kitchens in Hawaii [16]. In 1991, they reported that all food groups were either equal to (i.e., milk, vegetables, fruit, and meat), or above (i.e., bread), the recommended serves and there was no substantial difference in serve size; the serves per meal of bread was in fact 3.5 times greater than the recommended serves of bread per meal [16]. In contrast, our findings are somewhat different to those reported in previous Australian studies. For example, the serve sizes of meals produced by three different, non-commercial, MOW kitchens in Victoria were highly variable, and, moreover, the inconsistency in the serve sizes were not only observed between kitchens, but also within the same kitchen [9]. Similarly, a study in Northern Sydney found considerable differences in the serve sizes of numerous menu items from four separate MOW kitchens [17]. Notably, all Australian studies have indicated that serve sizes are greatly influenced by the subjective judgements of meal preparation staff (majority are volunteers) but also by differences in the serving utensils and the dimension of disposable containers that meals are packed into [9,17]. 

In this present study, for each of the STD and HEHP meals, we found significant differences in energy, protein and several micronutrients for the ‘measured’ compared to the ‘prescribed’. Despite these discrepancies, however, the ‘measured’ nutrient contents of each of the STD and HEHP meals were still in line with the HACC Guidelines [7,8]. We also found that the protein content of the STD compared with the HEHP meals was not substantially different (i.e., STD meals contained 42–63% of the RDI for protein and the HEHP contained 46–69%) although they did differ substantially with respect to their energy content (i.e., STD meals contained 21–39% of the RDI for energy, whereas the HEHP contained 29–55%). It is also important to note that the inconsistencies we found in the serve size of the dessert and main meal items, and, to a lesser extent, in the soups, could have affected the total amount of protein, energy and other micronutrients, within the overall meals. In fact, for the total meal, the inconsistencies represent an average deficit of 673 kJ and 11.7 g of protein for the STD and 409 kJ and 8.1 g for the HEHP meals. This might not be significant deficit for healthy clients of MOW, but for those who are recovering from illness or who are malnourished who typically rely on three to five meals per week from services like MOW, and this deficit could mean the difference between improving their nutrition status and overall health, or not. Moreover, Fogler-Levitt and colleagues reported that only 81% of home-delivered meals are actually eaten [18], and many studies conducted in Australia and overseas have documented that MOW clients—especially those who are malnourished—have insufficient food intake [19]. For example, a study of 124 MOW clients in Sydney found that 70% of male clients did not meet the dietary requirement (RDI) for protein, and around 30% did not meet the RDI for calcium, iron, thiamine and riboflavin [20]. Female clients also had similar consumption pattern; i.e., 70% of them did not meet the RDI for protein, while 80% and 40%, respectively, did not meet the RDI for iron and calcium [20]. Similarly, a study in Victoria that involved 124 MOW clients found that more than 55% of clients had protein and calcium intakes below the RDI [9]. Moreover, a study in Canada revealed that the average amount of energy and protein consumed from delivered meals by 150 MOW clients aged >75 years was 81 ± 18% and 82 ± 19%, respectively [18]. Collectively, our current findings, when taken together with the previous findings, indicate the need for some ongoing monitoring of meal services for consistency and accuracy. This may increase the likelihood that meal serve sizes better match recommendations, and the auditing of what is being delivered within a meal is easier than assessing what proportion of the meal clients are actually consuming; although we acknowledge both of these issues should ideally be evaluated as part of a yearly audit process for meal services such as MOW. This is possible through increased collaboration between food service professionals and accredited dietitians or nutrition departments who are seeking relevant training opportunities for dietetic students. 

When interpreting our data, it must be recognised that nutrient content of each meals were estimated using FoodWorks 3.01, which does not contain complete vitamin and mineral profiles for some (~10%) foods/ingredients that were present in the meal recipes. Additionally, the recipe for each menu item was entered as raw weight of food ingredients or condiments, and the “yield” function was applied to estimate the cooked weight after accounting for loss of water in the cooking process. However, due to limitations in software, there may be some discrepancies in final weight and nutrient content when compared to the values derived using other methods such as chemical analysis, which is not possible to perform during the study. Previous studies using chemical analysis indicated that certain food ingredients, such as fruit and vegetables, are prone to losing their nutrient content during the cooking process [21,22]. Retention of vitamin contents after the cooking process ranged between 0 (folic acid) and 94% (retinol), while mineral retention were between 63% (copper) and 96% (iron) [23]. Moreover, the core meals were prepared at a centralised kitchen rather than at local branches, where variability may be greater. A random audit of meals prepared at MOW kitchens from around South Australia, and the use of chemical analysis to most accurately determine the nutritional content of meals, would be very beneficial to get a better idea about the consistency of MOW meals in this state, and, hence, in Australia.

## 5. Conclusions

In conclusion, the measured serve sizes of the MOW meals were on average smaller than prescribed, although the differences were small. These findings also demonstrate that MOW meals currently meet the voluntary meal guidelines (set by the Australian Government) for energy, protein, vitamin C, calcium, phosphorus, iron and fibre, but fall ~10% short of the guidelines for potassium, magnesium and zinc. 

## Figures and Tables

**Table 1:** Food components and the prescribed serve size for each menu item within the Standard and High Energy and Protein fortified meals ^(a),(b)^.

Menu Item	Food Component	Prescribed Serve for STD Meal	Prescribed Serve for HEHP Meal
Soup	Stock with vegetables, noodles or grains or legumes, and meat or chicken	225 mL (cup)	225 mL (cup)
	Plus, 1 tablespoon of cream for fortification of HEHP soup		20 g
	Total volume	225 mL	245 mL
Main meal	Meat or Chicken or Fish	90 g	90 g
	Plus, potato or rice or pasta	90 g	90 g
	Plus, vegetables-yellow	65 g	65 g
	Plus, vegetables-green	65 g	65 g
	Plus, gravy or sauce	40 mL	40 mL
	Plus, 1 tablespoon of cream, 7 g butter, 10 g semi-matured grated cheese and fortified milk ^(c)^ to mashed potato		10–30 g
	Total weight	350 g	360–380 g
Dessert (two types of fortification, i.e., a or b)	Fruit based dessert (e.g., fruit crumble)	150–200 g	150–200 g
	Or, Canned fruit—6 apricot halves or 6–8 peach slices, or pear halves	150–200 g	150–200 g
	Or, pudding or cake	60 g	60 g
	Plus, (a) custard made with fortified milk ^(c)^	100 mL	100 mL
	Or, (b) Fortified milk ^(c)^ and cereal based dessert (e.g., rice pudding, bread and butter pudding) used in all MOW meals	150 g	150 g
	Plus, 1 tablespoon of cream for fortification of HEHP dessert		20 g
	Total weight	150–350 g	170–370 g

^(a)^ Prescribed serve sizes by MOW South Australia are based on voluntary guidelines recommended by the Australian Government and the State and Territory Governments jointly funded Home and Community Care (HACC) program to assist older people. These guidelines stipulate that each meal should provide at least one-third of recommended daily intakes (RDI) for energy, half of the RDI for protein and other vitamins and minerals, and two-thirds of the RDI for Vitamin C; ^(b)^ Prescribed serve sizes are in grams, or mL, and they have been calculated to provide RDIs for energy, protein and nutrients. These values represent the minimum cooked quantities exclusive of bone and, the nutrient calculations have allowed for shrinkage on meat, chicken and fish and other ingredients (e.g., cooking oil, sugar, salt, herbs and spices) used in cooking process were included in nutrient analysis; ^(c)^ Fortified milk is used for all MOW cooking and is achieved by adding 10 g of skim milk powder for every 100 mL of reduced fat (2% fat) high calcium milk; STD: standard meals, HEHP: high energy and protein fortified meals; nutrient content was analyzed using FoodWorks 3.01. MOW: Meals on Wheels.

**Table 2:** Prescribed and measured serve sizes and mean coefficient of variation of each menu item within the ‘Standard’ and ‘High Energy and Protein’ meals ^(a).^

Serve Size	STD			HEHP		
	Soup *N* = 20	Main *N* = 20	Dessert *N* = 20	Soup *N* = 20	Main *N* = 20	Dessert *N* = 20
Measured serve size (mL or g)	198 ± 28	372 ± 48	188 ± 55	211 ± 25	408 ± 36	245 ± 49
Prescribed serve size (mL or g)	265 ± 52	435 ± 56	237 ± 73	274 ± 58	456 ± 62	257 ± 73
CV of measured serves (%) ^(b)^	3	6	10	3	6	9
Difference (g)	−67	−63	−49	−63	−48	−12

^(a)^ all values represent the mean ± standard deviation (SD) per serve of the menu items from the meals that were actually weighed and prescribed during the summer menu meal audit (e.g., energy for measured item minus energy of prescribed item); These values represent the minimum cooked quantities exclusive of bone and, the nutrient calculations have allowed for shrinkage on meat, chicken, fish and other ingredients (e.g., cooking oil, sugar, salt, herbs and spices) used in cooking process were included in nutrient analysis; ^(b)^ CV (%) denotes the coefficient of variation which was calculated as the mean of the triplicate weights divided by the SD and multiplied by 100; STD: standard meals, HEHP: high energy and protein fortified meals. CV: coefficient of variation.

**Table 3:** Prescribed and measured nutrient contents per serve for soups, mains and desserts within the Standard and High Energy and Protein fortified meals ^(a).^

Nutrients	Prescribed			Measured			*p*-Values ^(b)^		
STD	Soup	Main	Dessert	Soup	Main	Dessert	Soup	Main	Dessert
Energy (kJ)	540 ± 132	1742 ± 320	1192 ± 516	433 ± 157	1387 ± 208	981 ± 468	0.024	0.001	0.18
Protein (g)	6.4 ± 2.6	32.5 ± 8.1	11.5 ± 5.0	5.0 ± 2.7	24.3 ± 7.1	9.4 ± 3.7	0.10	0.002	0.15
Carbohydrate (g)	10.3 ± 3.3	33.3 ± 11.0	38.5 ± 14.5	7.8 ± 2.6	29.8 ± 10.1	32.4 ± 14.9	0.011	0.30	0.2
Total Fat (g)	6.3 ± 3.0	14.7 ± 5.8	9.3 ± 9.7	5.3 ± 3.0	10.4 ± 4.5	7.3 ± 8.8	0.32	0.013	0.51
Fibre (g)	3.2 ± 1.7	9.0 ± 1.8	2.5 ± 1.4	2.5 ± 1.2	9.0 ± 2.2	1.9 ± 1.3	0.11	0.91	0.17
Vitamin C (mg)	21.2 ± 24.8	42.2 ± 21.8	7.3 ± 7.8	15.6 ± 17.0	40.6 ± 21.4	5.5 ± 6.3	0.41	0.81	0.43
Sodium (mg)	627.4 ± 1761.0	482.0 ± 328.9	189.1 ± 150.1	394.3 ± 1114.4	360.4 ± 218.6	142.4 ± 99.2	0.62	0.18	0.25
Potassium (mg)	385.5 ± 106.3	1270.8 ± 309.7	490.1 ± 169.2	296.1 ± 102.2	1089.3 ± 249.1	408.3 ± 150.5	0.01	0.048	0.11
Magnesium (mg)	25.6 ± 7.1	84.0 ± 18.2	37.8 ± 16.4	19.4 ± 5.6	71.8 ± 14.2	30.6 ± 12.5	0.004	0.022	0.13
Calcium (mg)	96.6 ± 50.5	141.4 ± 107.8	347.2 ± 165.4	81.3 ± 46.4	112.5 ± 86.0	296.4 ± 143.9	0.33	0.36	0.31
Phosporus (mg)	128.3 ± 49.5	448.0 ± 125.4	331.5 ± 186.6	103.3 ± 51.9	345.4 ± 101.8	267.3 ± 129.5	0.13	0.007	0.21
Iron (mg)	1.1 ± 0.6	3.6 ± 1.1	0.7 ± 0.4	0.8 ± 0.5	3.0 ± 0.9	0.6 ± 0.3	0.11	0.07	0.16
Zinc(mg)	0.8 ± 0.3	4.1 ± 1.9	1.1 ± 0.4	0.6 ± 0.2	3.2 ± 1.4	0.9 ± 0.3	0.018	0.11	0.10
HEHP	Soup	Main	Dessert	Soup	Main	Dessert	Soup	Main	Dessert
Energy (kJ)	666 ± 126	2155 ± 421	1472 ± 516	560 ± 138	1885 ± 307	1439 ± 498	0.015	0.026	0.84
Protein (g)	6.6 ± 2.5	32.8 ± 8.2	11.9 ± 5.0	5.3 ± 2.7	25.4 ± 6.8	11.6 ± 4.3	0.13	0.003	0.82
Carbohydrate (g)	10.5 ± 3.4	33.6 ± 11.1	38.9 ± 14.5	8.2 ± 2.5	32.0 ± 10	38.3 ± 14.9	0.023	0.63	0.90
Total Fat (g)	9.5 ± 1.7	25.6 ± 7.8	16.4 ± 9.7	8.4 ± 2.2	22.3 ± 6.0	15.9 ± 9.9	0.09	0.15	0.89
Fibre (g)	3.2 ± 1.7	9.0 ± 1.8	2.5 ± 1.4	2.5 ± 1.1	9.2 ± 2.2	2.3 ± 1.3	0.11	0.78	0.72
Vitamin C (mg)	21.2 ± 24.8	42.2 ± 21.8	7.3 ± 7.8	14.4 ± 17.3	41.6 ± 25.7	7.0 ± 7.3	0.32	0.93	0.93
Sodium (mg)	630.7 ± 1762.2	524.0 ± 328.0	196.3 ± 150.1	414.5 ± 1118.5	414.8 ± 224.0	179.9 ± 119.8	0.65	0.22	0.71
Potassium (mg)	396.4 ± 106.3	1285.8 ± 314.7	514.4 ± 169.2	313.6 ± 93.8	1145.0 ± 299.6	501.0 ± 141.8	0.013	0.16	0.79
Magnesium (mg)	26.1 ± 7.3	84.8 ± 18.5	39.0 ± 16.4	20.3 ± 5.2	74.7 ± 14.4	37.5 ± 13.4	0.006	0.06	0.75
Calcium (mg)	102.0 ± 46.0	148.2 ± 108.1	359.4 ± 165.4	91.5 ± 43.8	132.8 ± 91.4	354.1 ± 160.4	0.46	0.63	0.92
Phosporus (mg)	133.0 ± 46.3	565.1 ± 128.0	342.0 ± 186.6	111.0 ± 50.2	367.6 ± 104.3	326.0 ± 154.8	0.16	0.025	0.77
Iron (mg)	1.1 ± 0.65	3.6 ± 1.1	0.7 ± 0.4	0.8 ± 0.5	3.0 ± 0.8	0.7 ± 0.3	0.13	0.056	0.72
Zinc(mg)	0.8 ± 0.6	4.1 ± 1.9	1.2 ± 0.4	0.6 ± 0.2	3.2 ± 1.2	1.1 ± 0.4	0.022	0.08	0.77

^(a)^ Mean ± SD per serve of the menu items from the meals that were actually weighed during the summer menu meal audit; ^(b)^ Independent *t*-test; STD: standard meals, HEHP: high energy and protein fortified meals.

**Table 4:** Contribution of Standard and High Energy and Protein meals, as determined from the audit process, towards daily energy and protein requirements of older people.

Age and Gender	Estimated Energy Requirement (MJ) ^(a)^	Estimated Protein Requirement (g) ^(b)^	STD		HEHP	
			Energy Contribution (%) ^(c)^	Protein Contribution (%) ^(c)^	Energy Contribution (%) ^(c)^	Protein Contribution (%) ^(c)^
Male 51–70 years	9.5–12.1	76–91	23–29	42–51	32–41	46–56
Male >70 years	7.4–13.6	76–91	21–38	42–51	29–53	46–56
Female 51–70 years	7.6–9.6	61–73	29–37	53–63	41–51	58–69
Female >70 years	7.1–9.1	61–73	31–39	53–63	43–55	58–69

^(a)^ Based on the Australian Nutrient Reference Value (Reference weight for Male = 76 kg and Female = 61 kg); ^(b)^ Based on the PROTAGE Study group recommendation of 1.0–1.2 g protein/kg body weight (Reference weight for Male = 76 kg and Female = 61 kg); ^(c)^ Based on average values per serve of the STD and HEHP meals as measured during the summer menu meal audit; STD: standard meals, HEHP: high energy and protein fortified meals.
